# Evaluation of the relationship between the level of addiction and exhaled carbon monoxide levels with QT dispersion in smokers

**DOI:** 10.18332/tid/133053

**Published:** 2021-03-31

**Authors:** Gamze Keskin, Sibel Tunç Karaman, Okcan Basat

**Affiliations:** 1 Gaziosmanpaşa Training and Research Hospital, Department of Family Medicine, University of Health Sciences, Istanbul, Turkey

**Keywords:** arrhythmia, exhaled carbon monoxide, smoking, QT dispersion

## Abstract

**INTRODUCTION:**

Smoking increases the risk of arrhythmia. QT dispersion (QTd) is an important indicator for the determination of ventricular arrhythmia. In this study, we aimed to determine the arrhythmia risk by evaluating QTd in smokers and to assess the relationship between the level of nicotine addiction and carbon monoxide (CO) level in the expiratory air.

**METHODS:**

This study was designed as a single-center, cross-sectional study. Among the chronic smokers referred to the Smoking Cessation Clinic of a tertiary hospital between October 2019 and January 2020, all those who had no risk factors for cardiac arrhythmias, except smoking, were included in the study. Sociodemographic data and smoking characteristics of the participants were collected and exhaled CO levels were measured. QT intervals were measured in all leads by using a 12-lead standard electrocardiogram (ECG), and heart rate corrected QT (QTc) intervals, QT dispersion (QTd), and corrected QT dispersion (QTcd) were calculated.

**RESULTS:**

The mean age of the 250 patients was 37.2±9.3 years and the majority of patients (65%) were male. The mean amount of smoking was 25.74±16.03 packs/year and the mean value of CO was 12.36±7.06 ppm. The mean QTd was 23.83±13.12 ms, and the mean QTcd was 26.63±15.02 ms. A statistically significant relationship was found between QTd and QTcd and level of addiction, consumption of sticks/day and packs/year, and exhaled CO values (all p<0.001).

**CONCLUSIONS:**

It was found that as the level of addiction, cigarette use amount, exhaled CO levels, and BMI increased in smokers, QT dispersion and arrhythmia risk increased.

## INTRODUCTION

Smoking causes severe diseases and death and is one of the most crucial risk factors, particularly for cardiovascular system diseases^1^. A meta-analysis found a threefold increase in the relative risk of sudden cardiac death among current smokers^2^.

There are thousands of substances in cigarette smoke that are pharmacologically active, antigenic, carcinogenic, and addictive, including nicotine and carbon monoxide (CO)^3^. While CO poses a risk for cardiovascular system diseases by worsening hypoxia, another critical component of a cigarette, nicotine, increases the release of catecholamine, activates the sympathetic nervous system and closes the potassium channels located in the ventricular myocardium, and prolongs and delays repolarization^4,5^.

QT dispersion (QTd) (the difference between the maximum and minimum QT interval) is an easily measured electrocardiographic marker and indicates ventricular electrical instability and variable myocardial repolarization^6-8^. Thus, it is considered an important indicator of the severe risk of ventricular arrhythmia^6^. Values 40–50 ms are accepted as normal and values >65 ms are associated with an increased risk of severe ventricular arrhythmia^9,10^. In addition to the increase in a wide variety of cardiovascular diseases, QTd may also increase in non-cardiovascular diseases such as diabetes mellitus, rheumatological diseases, and vitamin D deficiency^11-13^.

Previous studies have investigated the effects of CO intoxication, smoking-related CO exposure, and acute and chronic smoking on QT dispersion. It has been shown that these factors may be associated with ventricular arrhythmia^14-16^. However, how QT dispersion is affected by the amount of smoking and addiction level has not been shown.

In this study, we aimed to determine the relationship between the amount of smoking, the level of nicotine dependence, and the exhaled CO level, an indicator of cigarette exposure, with QT dispersion, thus determining the risk of ventricular arrhythmia in smokers.

## METHODS

This study was designed as a single-center, cross-sectional study. Ethical permission to conduct this study was obtained from the Local Ethics Committee. (Approval No:146; 2 October 2019). The study was conducted under the principles of the Declaration of Helsinki. Written informed consent was obtained from all participants.

### Study population

All participants were selected from current chronic smokers who were referred for the first time to the Smoking Cessation Clinic of a tertiary hospital from October 2019 to January 2020. Two hundred and fifty people who had no risk factors for cardiac arrhythmia, except smoking, and agreed to participate, were included in the study. Those aged <18 years and those >65 years that had a history of known cardiovascular disease, diabetes mellitus, chronic pulmonary disease, and those under medication use (such as antibiotics, antihistamines, antiarrhythmic) that may affect QT distance, were excluded from the study. According to the G-power analysis made in line with the reference studies, the minimum number of participants required for the study was 146 with a 95% CI.

### Data collection tools

#### Patient information form

A patient information form was formulated, which included the participants’ sociodemographic characteristics (age, gender, marital status, educational level), smoking amounts (sticks/day and packs/year), medical history, BMI (Body Mass Index, kg/m^2^), and exhaled CO measurement.

#### Fagerström Test for Nicotine Dependence (FTND)

The Fagerström test for nicotine dependence (FTND)^17^ and the Turkish validity and reliability study conducted by Uysal et al.^18^ were used to measure the degree of nicotine dependence of the participants. The dependence level was categorized according to the following FTND scores: 0–2 very low; 3–4 low; 5 moderate; 6–7 high; and 8–10 very high.

#### Exhaled CO measurement

Exhaled CO is increased in smokers and is a biomarker frequently used in the diagnosis, treatment, and follow-up stages of cigarette dependence^19^. Exhaled CO measurements (in parts per million, ppm) were performed by authorized healthcare staff using piCO + Smokerlyzer (Bedford Scientific, Maidstone, UK, 2016) devices.

#### Electrocardiographic examination and evaluated parameters

The 12-lead ECG was performed with the Nihon Kohden brand ECG-1350K model device, at a speed of 25 mm/s and 10 mm/mV, while the participants were at rest in the supine position. All the patients had sinus rhythm. Heart rate and QT intervals of the patients were measured manually by a single investigator on ECG with a ruler and magnifying glass, and no computer program was used. The QT interval between the start of the QRS complex and the end of the T wave was measured in milliseconds. QT intervals in all leads were evaluated by using 12-lead electrocardiograms. Heart rate corrected QT (QTc) intervals, QTc minimum, and QTc maximum values were then calculated by using Bazett’s formula [QTc=QT/√(RR/1s) ]^20^. QTd was defined by calculating the difference between the longest and shortest QT intervals. The corrected QT dispersion (QTcd) was defined by calculating the difference between the longest and shortest QTc intervals.

### Statistical analysis

The IBM SPSS Statistics 22 program was used for statistical analysis. The compliance of the parameters to normal distribution was evaluated with the Shapiro-Wilk test. In addition to descriptive statistical methods (mean, standard deviation, frequency) in more than two group comparisons, one-way ANOVA was used when numerical variables showed normal distribution, and the Kruskal-Wallis test was used when there was no normal distribution. Mann-Whitney U test was used to determine the group that caused the difference. Mann-Whitney U test evaluated the comparisons of normally distributed parameters between two groups. Pearson’s correlation analysis was performed to examine the relationships between parameters that conform to a normal distribution, and Spearman’s rho correlation analysis was used to examine relationships between parameters that did not conform to a normal distribution. Significance was considered at the level of p<0.05.

## RESULTS

The mean age of the 250 participants included in the study was 37.2±9.3 years, and 62% were male (n=155). The mean amount of smoking was 25.74±16.03 packs/year and the mean value of exhaled CO was 12.36±7.06 ppm. The mean BMI was 25.81±4.15 kg/m^2^. Table 1 presents the sociodemographic and smoking characteristics of the participants.

**Table 1:** Sociodemographic and smoking characteristics of the study population (N=250)

*Characteristics*	*Range*	*Mean±SD*
**Age** (years)	18–65	37.2±9.3
**BMI** (kg/m^2^)	16.53–41.77	25.81±4.15
**Smoking amount** (packs/year)	2–105	25.74±16.03
**Smoking rate** (sticks/day)	5–90	27.34±11.5
**FTND score**	0–10	6.64±2.22
**Exhaled CO level** (ppm)	0–32	12.36±7.06

***Characteristics***	***Categories***	***n (%)***

**Gender**	Female	95 (38.0)
	Male	155 (62.0)
**Alcohol use**	No	183 (73.2)
	Yes	67 (26.8)
**Smoking rate** (sticks/day)	≤10	15 (6.0)
	11–20	90 (36.0)
	21–30	82 (32.8)
	≥31	63 (25.2)
**Level of dependence**	Very low	13 (5.2)
	Low	30 (12.0)
	Moderate	25 (10.0)
	High	79 (31.6)
	Very high	103 (41.2)

BMI: body mass index (kg/m^2^). FTND: Fagerström test for nicotine dependence. CO: carbon monoxide.

The mean QTd was 23.83±13.12 ms, and the mean QTcd was 26.63±15.02 ms. The electrocardiographic findings of the participants are summarized in Table 2.

**Table 2:** Electrocardiographic findings of the study population (N=250)

*Measurements*	*Range*	*Mean±SD*
HR (beats/min)	52–112	74.57±10.66
QT max (ms)	300–440	367.19±26.26
QT min (ms)	275–420	343.36±26.93
QTd (ms)	4–64	23.83±13.12
QTc max (ms)	293–503	406.67±26.98
QTc min (ms)	279–467	380.04±26.51
QTcd (ms)	4–85	26.63±15.02

HR: heart rate. QT max: QT maximum. QTc max: corrected QT maximum. QT min: QT minimum. QTc min: corrected QT minimum. QTd: QT dispersion. QTcd: corrected QT dispersion.

Exhaled CO values of male smokers were significantly higher (p=0.005). It was found that as the number of daily cigarettes increased, exhaled CO values increased (p<0.001).

Table 3 presents differences in QTd and QTcd according to the smoking and sociodemographic characteristics. The QTd and QTcd values of female smokers were significantly lower than those of the male smokers (p=0.009; p=0.013, respectively). A significant difference was found regarding the number of cigarettes smoked daily and QTd and QTcd values (p<0.001; p<0.001, respectively). The QTd and QTcd values of those who smoke ≥31 cigarettes per day were significantly higher than those who smoked less (p<0.001; p<0.001, respectively).

**Table 3:** Relationship between QTd and QTcd and smoking and sociodemographic characteristics (N=250)

*Characteristics*		*QTd*	*QTcd*
**Age**	Spearman rho	0.111	0.093
	p	0.081	0.142
**BMI**	Spearman rho	0.162	0.163
	p	0.010*	0.010*
**Gender**	Female	21.22 (12.12)	23.67 (13.6)
	Male	25.43 (13.48)	28.44 (15.6)
	p^a^	0.009*	0.013*
**Total amount of smoking (pack/years)**	Spearman rho^c^	0.328	0.312
	p	<0.001*	<0.001*
**Cigarettes per day**	≤10	10.27±4.38	11.67±4.81
	11–20	20.11±11.54	22.44±13.4
	21–30	26.34±12.41	29.73±14.7
	≥31	29.11±13.89	32.13±15.41
	p^b^	<0.001*	<0.001*
	Spearman rho	0.396	0.388
**FTND scores**	Spearman rho^c^	0.539	0.547
	p	<0.001*	<0.001*
**Exhaled CO levels**	Spearman rho^c^	0.837	0.838
	p	<0.001*	<0.001*
**Alcohol use**	No	22.89±13.06	25.63±15.03
	Yes	26.4±13.01	29.34±14.77
	p^a^	0.040*	0.048*

Data presented as Mean±SD.

a Mann-Whitney U test.

b Kruskal-Wallis test Pearson correlation analysis.

c Spearman rho correlation analysis.

* Indicates statistical significance.

BMI: body mass index (kg/m^2^). CO: carbon monoxide. FTND: Fagerström test for nicotine dependence. QTd: QT dispersion. QTcd: corrected QT dispersion.

As the scores from FTND and amount of cigarette consumption of the participants in terms of sticks/day and packs/year increased, QTd and QTcd also increased significantly (all p<0.001). We also observed that as BMI values increased, QTd and QTcd increased significantly (p=0.010 and p=0.010, respectively). A positive and significant relationship was present between QTd and QTcd with exhaled CO values (p<0.001; p<0.001, respectively) (Table 3).

Table 4 shows the differences in ECG findings concerning addiction levels. No significant relationship was observed between the addiction levels and the longest and the shortest corrected QT values (p>0.05 for both). However, a statistically significant difference was found between addiction levels regarding QTd and QTcd values (p<0.001; p<0.001, respectively).

**Table 4:** Evaluation of the relationship between FTND addiction levels and ECG parameters (N=250)

*Level of addiction (FTND score)*	*QTc max*	*QTc min*	*QTd*	*QTcd*
Very low (0–2)	410.15±32.54	398.31±35.62	10.85±8.23	11.85±8.40
Low (3–4)	395.93±24.27	381.07±25.12	13.60±6.77	14.87±7.22
Moderate (5)	402.52±34.67	378.64±33.47	21.68±9.98	23.88±11.26
High (6–7)	405.85±25.58	381.62±25.38	21.85±10.87	24.23±12.13
Very high (8–10)	410.99±25.35	376.56±23.95	30.50±13.56	34.43±15.78
p	0.081	0.079	<0.001*	<0.001*

Data presented as Mean±SD.

* p indicates statistical significance.

QTc max: corrected QT maximum. QTc min: corrected QT minimum. QTd: QT dispersion. QTcd: corrected QT dispersion.

## DISCUSSION

In the present study, the relationship between smoking and some ECG changes pertaining to increased cardiac arrhythmia risk was investigated, and it was shown that as the level of addiction, the number of cigarettes consumed, exhaled CO levels, and BMI increased, the QT dispersion, which is essential for determining the risk of ventricular arrhythmia, increased.

Smoking is the most crucial, preventable, and modifiable risk factor for cardiovascular system diseases and can cause severe ECG changes that may be associated with ventricular arrhythmia. Especially, CO and nicotine play an important role in these effects of cigarettes on the cardiovascular system^4^.

In previous studies, the effect of QTd on mortality in the general population was investigated, and it was found that increased QTd raised the risk of cardiac events and mortality^21-23^. The probable prognostic significance of the QTd was evaluated in the preliminary report of the WOSCOPS study. Middleaged men without a history of previous myocardial infarction were examined, and increased future myocardial infarction risk has been demonstrated in those who have QTd >44 ms^22^. In another study, healthy volunteers aged >55 years were followed up for four years, and those with QTcd of ≥60 ms were found to have an increased risk of total mortality, cardiac death, and sudden death^21^. In the study made by Malik et al.^23^ in 2000, which evaluated >40 studies conducted in the last three years related to QTd, the mean QTd values were 33.4 ms in healthy individuals, 59.2 ms in patients with previous myocardial infarction, 70.9 ms in patients with acute myocardial infarction, and 83.2 ms in patients with long QT syndrome^23^.

In our study, evaluating only chronic smokers, the QTd values ranged 4–64 ms. Although the mean QTd value of smokers was observed to be within normal limits, as the level of addiction, cigarette consumption, and exhaled CO level increased, an increase in QTd was observed, proving the increased risk of arrhythmia with smoking. The highest QTd value we detected in the participants was 64 ms, and this value was higher than for patients who previously had a myocardial infarction.

In the literature, there are studies evaluating the effects of acute and chronic smoking on QT interval and QTd, but different results have been reported from these studies^16,24-28^. Ileri et al.^25^ compared habitual smokers and non-smokers in their study, and chronic cigarette smoking was found to prolong QT interval in healthy people, and habitual smokers had significantly increased QTcd compared to nonsmokers. Although habitual smoking was assessed, the subjects were also assessed under the effect of some acute smoking, because according to the study protocol, they had to smoke 5 cigarettes before the QT measurements were obtained^25^.

In a study investigating the effect of acute smoking on QTd, it was observed that QTd and QTcd increased significantly even after a single cigarette in both healthy smokers and non-smokers, which means that even in chronic smokers, there is no cardiac adaptation and tolerance to the negative effects of smoking, regardless of smoking status; therefore, even one cigarette is thought to increase the risk of arrhythmia^16^.

In the study conducted by Taşolar et al.^26^ comparing healthy chronic smokers and non-smokers, the mean QTd in smokers was 34.2±8.4 ms, and the mean QTcd was calculated as 37.3±8.9 ms. In addition to the fact that these parameters were higher in smokers than non-smokers, it was determined that the QTd increased as the degree of smoking increased^26^. In our study, QTd and QTcd values were found approximately 10 ms longer than in that study.

In addition to the findings of these studies, there was a significant difference between addiction levels in terms of QTd and QTcd values in our study. We found that QT dispersion increased with the increased level of addiction.

In contrast to these studies, Dilaveris et al.^27^ found that only corrected QT interval was prolonged in smokers; however, the QTd did not differ between smokers and non-smokers^27^. Similarly, Karakaya et al.^28^ reported that although a single-dose smokinginduced sympathetic activity, it did not affect QTd.

To our knowledge, even though there are studies in the literature evaluating the relationship between smoking status and QTd and QTcd parameters, the number of studies making a comparison with the level of nicotine addiction is limited. More extensive studies are needed in this area.

Besides the effects of cigarette smoking, in a study evaluating the ECG parameters before and after hookah smoking, the average QTd and QTcd values of the participants measured after the hookah smoking increased statistically significantly from the values obtained before the hookah smoking^14^. Although the average QTd and QTcd values we measured in our study were found to be lower than the values obtained in that study, it is thought that the differences may be due to measurement methods; it is possible that the acute effects of hookah are more significant than those of cigarettes.

CO level, the indicator of cigarette exposure, is related to increased QT dispersion. In previous studies, it has been reported that QT interval and QT dispersion increase in patients with acute CO intoxication^29,30^. Atesçelik et al.^29^ found that both QTd and corrected QTd were longer in CO intoxicated patients than the control group and decreased one week later after intoxication.

We found a significant relationship between the value of CO measured from expiratory air and QT dispersion and concluded that although it was not high enough to cause intoxication, regardless of the measurement technique, CO increased the risk of ventricular arrhythmia. However, exhaled CO measurements have some disadvantages. Since the half-life of CO is short, it is more significant in terms of recent exposure. Also, the measured level may be affected due to environmental tobacco exposure and/or non-cigarette CO sources. Moreover, the QT dispersion may vary for 24 hours. The electrocardiograms of the participants also vary depending on the time of outpatient clinic applications.

## CONCLUSIONS

This study found a significant relationship between the level of addiction, amount of smoking and the exhaled CO levels, and QT dispersion. Our study showed that the risk of arrhythmia increased as the level of addiction increased in smokers. Evaluations in terms of QTd with ECG, are simple and applicable to all smokers and vital in determining the risk of arrhythmia.
